# Cortical representation of musical pitch in event-related potentials

**DOI:** 10.1007/s13534-023-00274-y

**Published:** 2023-04-13

**Authors:** Taehyoung Kim, Miyoung Chung, Eunju Jeong, Yang Seok Cho, Oh-Sang Kwon, Sung-Phil Kim

**Affiliations:** 1 Department of Biomedical Engineering, Ulsan National Institute of Science and Technology, Ulsan, Republic of Korea; 2 Department of Music and Science for Clinical Practice, College of Interdisciplinary Industrial Studies, Hanyang University, Seoul, Republic of Korea; 3 School of Psychology, Korea University, Seoul, Republic of Korea

**Keywords:** Pitch, Event-related potential, Musical training, Hemispheric differences, Frontotemporal cortex

## Abstract

Neural coding of auditory stimulus frequency is well-documented; however, the cortical signals and perceptual correlates of pitch have not yet been comprehensively investigated. This study examined the temporal patterns of event-related potentials (ERP) in response to single tones of pitch chroma, with an assumption that these patterns would be more prominent in musically-trained individuals than in non-musically-trained individuals. Participants with and without musical training (N = 20) were presented with seven notes on the C major scale (C4, D4, E4, F4, G4, A4, and B4), and whole-brain activities were recorded. A linear regression analysis between the ERP amplitude and the seven notes showed that the ERP amplitude increased or decreased as the frequency of the pitch increased. Remarkably, these linear correlations were anti-symmetric between the hemispheres. Specifically, we found that ERP amplitudes of the left and right frontotemporal areas decreased and increased, respectively, as the pitch frequency increased. Although linear slopes were significant in both groups, the musically-trained group exhibited marginally steeper slope, and their ERP amplitudes were most discriminant for frequency of tone of pitch at earlier latency than in the non-musically-trained group (~ 460 ms vs ~ 630 ms after stimulus onset). Thus, the ERP amplitudes in frontotemporal areas varied according to the pitch frequency, with the musically-trained participants demonstrating a wider range of amplitudes and inter-hemispheric anti-symmetric patterns. Our findings may provide new insights on cortical processing of musical pitch, revealing anti-symmetric processing of musical pitch between hemispheres, which appears to be more pronounced in musically-trained people.

**Supplementary Information:**

The online version contains supplementary material available at 10.1007/s13534-023-00274-y.

## Introduction

Pitch is the perceptual experience of the fundamental frequency of a sound [49]. Humans use pitch information for various communication purposes; namely, in the context of music, speech, and other social interactions. For example, pitch is vital in creating diverse musical structures such as intervals, contours, and harmonic chords, which induce a wide range of emotional responses [15, 55]. In addition, pitch can signal different meanings for monosyllabic words in certain tone languages such as Mandarin Chinese [37]. Thus, although pitch may be considered a universal means of communication, pitch perception varies across individuals, both inherently and by experience, as reported by previous studies investigating the increase in pitch sensitivity through experience and learning [7, 43, 44, 67].

The neural coding or processing of sound frequency has been identified using two schemes: place and temporal coding. Place coding is based on spatial position in the basilar membrane, whereas temporal coding is based on timing information and phase synchrony of neuronal spikes [48]. A tonotopic map of the basilar membrane maintained in the primary auditory cortex (A1) correlates with the encoding of the fundamental frequency of a sound [3, 51]. Recent studies have suggested a hybrid spatiotemporal neural processing model that combines both place and temporal coding [9, 11]. In addition, frequency-following responses measured by electroencephalography (EEG) reflect brain stem activity that is phase-locked to sound waves [13] and represents the sound frequency, timbre, and harmonics [39].

Pitch distinction is the most basic process when listening to music. Several previous studies have attempted to find the neural correlates of different pitches in the Western tonal context. Bidelman and Grall reported that neural responses to pitch emerge within 150 ms of sound onset, which follows Western harmony rules rather than those of simple acoustic attributes [4]. Sauve et al. [62] reported that neural marker amplitudes and latencies were significantly correlated with similar magnitudes of pitch height and goodness-of-fit ratings [62]. This is distinguished by the division between pitch height and tonal hierarchy correlating with early (100–200 ms) and late (200–1000 ms) neural markers, respectively (Sankaran et al. 2020) [53]. Moreover, Kim and Knösche [34] demonstrated a pathway, which is relevant to the absolute pitch process, from the primary auditory cortex (PAC) to the ventrolateral prefrontal cortex (VPC) [34]. Although these findings reveal the brain’s processing mechanisms for pitch and pitch height in a tonal context, the neural correlates of each single frequency in pitch are relatively unknown. Moreover, several studies have reported brain activation that exhibit stronger responses to musical pitch than to non-musical pitch in the lateral and medial Heschl’s gyrus [25, 32].

Hemispheric difference is a marked property of neural pitch processing. Previous research has indicated that the right auditory cortex is more predominantly involved in pitch processing than the left auditory cortex. Patients who underwent right auditory cortex resection demonstrated poorer performance in pitch direction detection than those who underwent left auditory cortex resection (Johnsrude et al. 2000). In addition, more accurate decoding of melodic components from neural activity was observed in the right hemisphere than in the left hemisphere [1]. Early differences in the right-sided perisylvian brain regions reflect auditory tone categorization [8]. In addition, there were several crucial findings of hemispheric dominance in the left dorsolateral prefrontal cortex [18, 56]. Musicians with absolute pitch have left-hemisphere activation, which is associated with enhanced vocal pitch error detection [2].

Pitch perception possibly involves hierarchical auditory stimuli processing; hence, neural representations of pitch can be found over multiple cortical areas such as inferior frontal gyrus (IFG), intraparietal sulcus (IPS), and superior/middle temporal gyrus (STG/MTG) including the A1 (Merrill et al. 2012; Tsai and Li 2019) [28, 50]. Studies on absolute pitch have implicated a network of the dorsolateral frontal cortex and bilateral temporal planum in its neural processing [28]. In particular, the right inferior frontal gyrus (IFG) volume is associated with individual differences in pitch discrimination [50]. Furthermore, congenital amusia is associated with white matter in the IFG [41]. These findings suggest that neural representations of pitch may possibly emerge over several cortical areas, thereby providing a motive to examine a wide range of areas to find pitch-related neural responses.

Musical expertise is an additional factor influencing the neural correlates of pitch. Differences in pitch discrimination resulting from musical training have been reported in several studies [22, 24, 26]. Musical training increases brain plasticity associated with musical performance [59, 60, 67], hence, it is possible that musical training similarly affects the neural representations of pitch. Previous studies reported that neural representations of pitch in relevant subcortical and cortical areas could be enhanced by music-related experiences of multiple levels of the auditory pathway [38]. Neural correlates of pitch would involve temporal dynamic changes in response to a given musical stimulus. For example, the detection of a sound would generate an auditory evoked potential (AEP) followed by neural responses corresponding to the pitch discrimination information; all these processes would concurrently occur in hundreds of milliseconds [66]. Hence, investigation of the neural correlates of pitch may require a high temporal resolution to discover such dynamics, thereby needing measurements with a high temporal resolution, such as an EEG or a magnetoencephalogram.

In particular, examination of the temporal patterns of event-related potentials (ERP) across whole brain areas would reveal the characteristics of neural representations of pitch. The temporal patterns of ERPs with a high temporal resolution can signify the temporal dynamics of the neural representations of pitch. In addition, whole-brain measurements of ERPs would reveal the different bilateral hemispheric representation of pitch, indicated by hemispheric asymmetry. In addition, temporal patterns of ERPs in different cortical areas would help identify areas wherein neural representations of pitch are more pronounced. Furthermore, neural representations of pitch in ERPs could differ according to musical training, as reported by previous studies on the effects of musical expertise on ERP components [35, 47].

This study aimed to identify neural correlates of pitch in human brain signals. In particular, we intended to answer the four following interrelated questions: first, whether it is possible to find a neural correlate of pitch in human ERPs; second, whether such neural correlates of pitch in ERPs are represented differently between hemispheres according to previous findings about differential neural processing of musical pitch between the hemispheres(see above); third, identifying which brain areas reveals neural correlates of pitch; and fourth, whether neural correlates of pitch in ERPs are more salient in musically-trained individuals than in non-musically-trained individuals based on previous finding indicating the effects of musical training on behavioral and neural processing of pitch.

A key distinction of the present study is that we focused on neural responses to individual tones with different frequencies of pitch rather than considering tonal hierarchy or tonal context. This study questions how brain activity discriminatively represents a single frequency of pitch. Furthermore, we assumed that musical training might enhance discriminative representations of a single frequency of pitch; this is different from previous studies, which investigated changes in neural responses to an identical tone presented in different musical tonal contexts. We assumed that individuals can distinguish relative differences (and possibly, relative frequency levels) between individual frequencies of pitch by comparing the given pitches to each other even if they were not in any tonal context.

To address these questions, the cortical activity of healthy participants was measured using EEG, and the ERPs in response to randomly presented sound pitches on a C major scale (i.e., C4, D4, E4, F4, G4, A4, and B4) were analyzed. Subsequently, ERP patterns correlated with the frequency of pitch were explored, and the possible asymmetry of the patterns between the left and right hemispheres was examined. Further, differences in ERP patterns between musically-trained and non-musically-trained groups were assessed to evaluate the effects of musical training.

## Methods and materials

### Participants

This study recruited 20 university undergraduate students; 10 participants with a minimum of 3 years of formal musical training (musically-trained [MT] group; male = 5, female = 5; average age = 24.2 ± 1.8 years) and 10 participants without musical training (non-trained [NT] group; male = 6, female = 4; average age = 25.5 ± 2.0 years). All musically-trained participants were able to play the piano and had the ability to accurately distinguish individual notes by listening to the piano scales. None of the participants reported any abnormalities related to auditory function or neurological disorders. All participants were right-handed.

### Ethics approval statement

#### Research involving human participants

This study was approved by the Institutional Review Board (IRB) of the Ulsan National Institute of Science and Technology (UNISTIRB-20-22-A) and conformed to the tenets of the Declaration of Helsinki.

#### Informed consent

Written informed consent was obtained from all participants prior to the experiment; participants were remunerated for their participation in the study.

### Stimuli

**Fig. 1:**
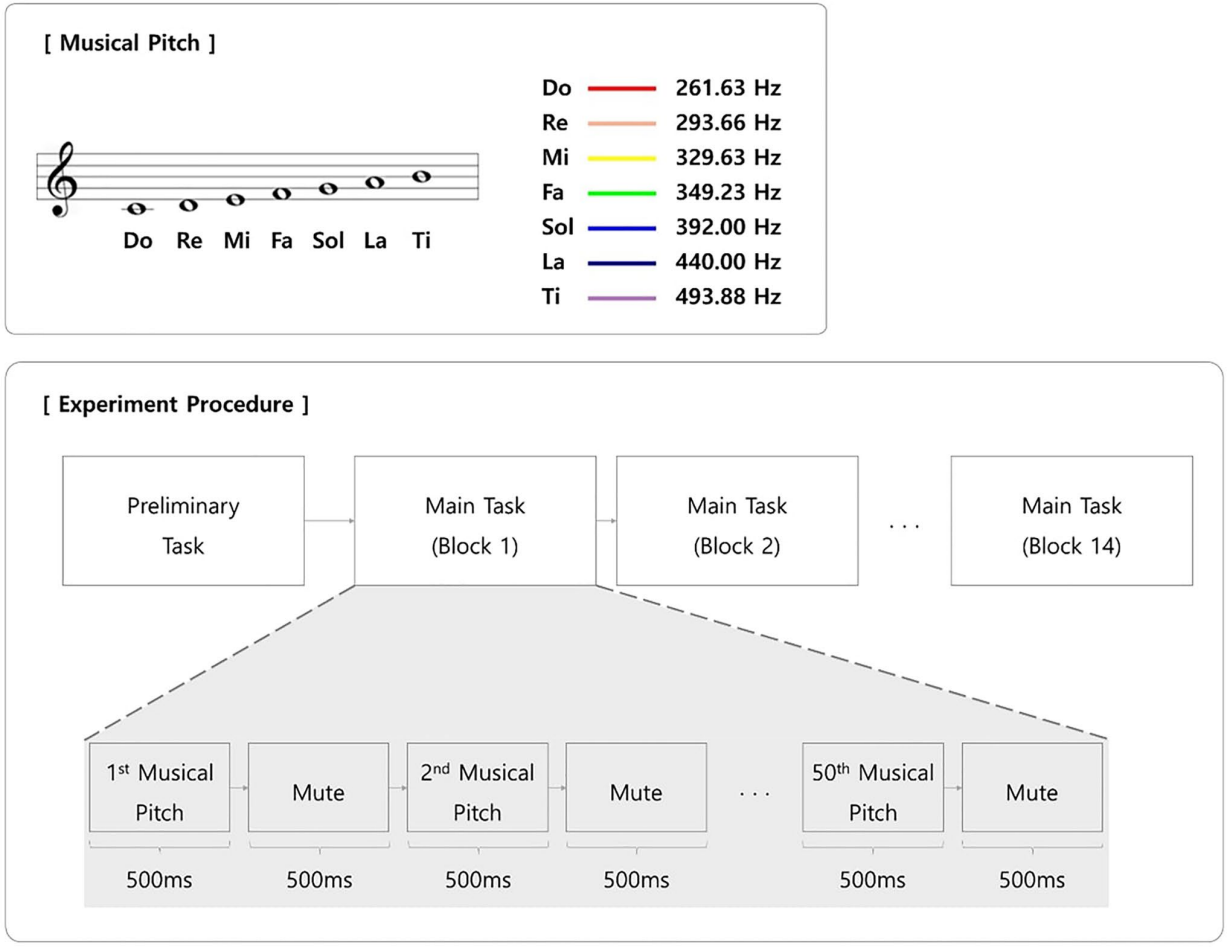
Experimental procedure. Before the main task, participants perform a preliminary task. In this task, participants listen stimulus from C4–B4 in ascending and descending order twice. The experiment comprises fourteen blocks of pitch perception. For each block, one of the seven pitches—“Do, Re, Mi, Fa, Sol, La and Ti” as illustrated in the inserted score sheet—is designated as a target pitch and participants are asked to count the number of presentations (the color of the block in the figure signifies a target pitch presented in the legend; e.g., the target pitch of block 1 is “La”). Each pitch is randomly designated as target, twice during the fourteen blocks. In a block, a sound stimulus with pure tone with randomly selected pitch is presented for 500 ms, followed by a 500-ms silent period. This sound presentation is repeated 50 times. The presentation of each pitch is pseudo-randomized to ensure that each pitch is exactly presented 100 times during the experiment

A set of auditory stimuli corresponding to the seven notes of the C major scale (Fig. 1A) was prepared. These auditory stimuli were synthesized as grand piano tones (C4:261.63 Hz, D4:293.66 Hz, E4:329.63 Hz, F4:349.23 Hz, G4:392 Hz, A4:440 Hz, and B4:493.88 Hz) using Logic pro with a MIDI device (M-audio Keystation 61, USA). All stimuli had the same sound intensity (90 dB) and sound envelope components (500 ms length) without any fade-in or fade-out effect. All stimuli had the same value of attack, decay, sustain, and release because we ensured that every note in Logic Pro was made exactly in the same manner. Participants listened to a stimulus through earphones (Panasonic RP-HV094, Japan) in an electromagnetically shielded space. Stimuli were provided binaurally (stereo).

### Task

In this study, all participants performed a pre-task before the main task. The pre-task was a pitch identification exercise designed to screen musically-trained participants in order to exclude those who were originally recruited in the MT group based on their report of musical experience, although they may be unable to identify pitch; hence, it was used to assess the participant’s ability to identify absolute pitch. However, the pre-task is unrelated to the main experiment in this study; therefore, the behavioral and neural data recorded during this task was not used in the analysis. In the pre-task, participants were informed about the aforementioned seven notes in the C major scale. Subsequently, they randomly listened to one note and verbally answered the corresponding notes. This test was repeated five times for each participant. All MT group participants perfectly identified the musical pitches. Conversely, the NT group participants failed to identify the musical pitches and demonstrated an almost random identification. For convenience, the sound presented in the pre-task was produced by the grand piano simulator in the Garage Band App of iPad Air (2nd generation).

The main task began with a preliminary presentation of each auditory stimulus twice to the participants. Each stimulus was presented for 500 ms, followed by a 500-ms silent period from C4–B4 in ascending and descending order. This presentation was intended to familiarize the participants with the musical scale of the pitches that they would hear in the subsequent task, which comprised one practice block followed by 14 main blocks that were similarly designed. Each block contained 50 trials of stimulus presentation. At the beginning of each block, participants were presented a target note in text form on the display screen in front of them. Subsequently, they were instructed to count the number of times the target note was presented in the block. These instructions were provided to maintain participants’ engagement in the task. The target note in text form appeared until the participants pressed any key on the keyboard to start the block. Each of the 50 trials serially proceeded without a break. In each trial, one of the seven auditory stimuli was randomly presented to each participant. The duration of a single trial was 1,000 ms; beginning with the presentation of a stimulus for 500 ms, followed by a 500-ms silent period (Fig. 1). A piano keyboard illustrating the seven notes was continuously presented on the display screen throughout the 50 trials as a visual aid. The presentation order of the stimuli during the trials and blocks was pseudo-randomized to ensure that every stimulus was presented equally; each stimulus was presented exactly 100 times during the main blocks. Participants were asked to report the number of times the target note was presented at the end of each block. Each note was assigned as a target twice during the main blocks, and the assignment of targets was randomized across participants. After the block ended, the participants pressed any key on the keyboard when to proceed to the next block. Data from the practice block were excluded from the analysis. The experimental protocol was created using MATLAB Psychtoolbox (2019b, V3.0.16, Mathworks, Inc., Natick, MA, USA).

The objective of asking the participants to count target pitch presentation in the main task was to help them carefully listen to each single stimulus in the block. However, this counting task on ERP patterns could have resulted in possible side effects induced by complex cognitive processes such as working memory, pitch-class identification, and arithmetic counting process. To address this, first, we analyzed ERPs in response to non-target notes only to avoid a possible effect of the counting process on target notes. Moreover, other possible cognitive processes may work equally for all non-target trials; hence, we assumed that a possible effect of these processes on ERPs could be common among all the musical notes and consequently, remain undifferentiated.

### EEG recordings

Scalp EEG signals were recorded from 31 active wet electrodes (FP1, FPz, FP2, F7, F3, Fz, F4, F8, FC5, FC1, FCz, FC2, FC6, T7, C3, Cz, C4, CP5, T8, CP1, CPz, CP2, CP6, P7, P3, Pz, P4, P8, O1, Oz, and O2) using a standard EEG cap placed on the scalp following the 10–20 system of the American Clinical Neurophysiology Society Guideline 2. EEG signals were amplified using a commercially available amplifier (ActiCHamp, Brain Products GmBH, Germany). The sampling rate was 500 Hz. Ground and reference electrodes were attached to the left and right mastoids, respectively. Contact impedance was maintained at less than 10 K Ω. The reference and ground channels were located in the right and left mastoids, respectively.

### EEG analysis

#### Preprocessing

Line noise was eliminated using a notch filter at 60 Hz with a 2-Hz bandwidth. Subsequently, the EEG signals were filtered using a bandpass filter with a low cutoff frequency of 1 Hz and a high cutoff frequency of 50 Hz. Furthermore, bad EEG channels were detected and eliminated by inspecting each channel to check if > 70% of all other channels demonstrated a cross-correlation < 0.4 with that channel [6]. This bad-channel detection process eliminated 3.45 channels on average (std = 3.08) across participants. The eliminated bad channels were replaced by virtual EEG signals synthesized via spherical interpolation. Subsequently, the EEG signals were re-referenced using the surface Laplacian method to establish a local relationship between the scalp potentials and the underlying source-level potential [10]. The artifacts were eliminated using the artifact subspace reconstruction (ASR) method. The cut-off parameter used in the ASR was set to 30, following the guidelines suggested in a previous study [12]. EEG preprocessing was performed using the EEGLAB Toolbox of the Swartz Center for Computational Neuroscience (SCCN) [17] and a pipeline developed by Bigdely-Shamlo et al. [6].

#### ERP analysis

An epoch for the ERP analysis was defined as 100 ms–800 ms (before–after stimulus onset) in each trial. ERP waveforms were further explored to identify discriminative ERP features according to pitch. Therefore, we sought a specific time window in which ERP amplitudes would be most distinguished between pitches for all channels. To avoid the circular fallacy problem (i.e., the double-dipping problem), the first half of the trials was used to find the optimal window, whereas the latter half was used for subsequent analyses. We set the window size to 100 ms, with a 90-ms overlap. The discrimination of pitch amplitudes in each window was assessed by calculating the separability of ERP amplitudes by pitch. Separability was calculated based on the Davies-Bouldin index (DBI) [16]. Unlike conventional DBI, which finds an optimal number of clusters with the maximum ratio of inter-cluster distance to intra-cluster distance, in our study separability was calculated as the average ratio of inter-pitch distance to intra-pitch distance between every pair of pitch, as follows:

1 $$\mathrm{Separability}\frac{1}{C(\mathrm{7,2})}\sum_{i}\sum_{j}\frac{d({c}_{i},{c}_{j})}{{\sigma }_{i}+{\sigma }_{j}}, j\ne i ,$$

where σ_*i*_ and σ_*j*_ are the standard deviations of the ERP amplitudes within a given window for the *i*-th and *j*-th pitch, respectively, and $d({c}_{i},{c}_{j})$ is the inter-pitch distance. This distance was calculated as the difference between the median values of the *i*-th and *j*-th pitch.

After the time window for the analysis was determined as outlined above, one-way analysis of variance (ANOVA) was used to select EEG channels that demonstrated significant differences in ERP amplitudes among the pitches in that window, after checking the normality of the sample data with the Kolmogorov–Smirnov test. If the mean ERP amplitude in the window at a given channel revealed a significant difference (p < 0.05) in any of the two groups (NT and MT), that particular channel was included in the set of selected channels. After collecting channels with significant differences across pitches, the set of selected channels was completed by adding channels that were bilaterally symmetric to one of the collected channels yet did not reveal significant differences across pitches. For example, if channel F4 was selected because of a significant difference across pitches though F3 was not, F3 was added to the set because it was bilaterally symmetric to F4. Thus, a set of bilaterally symmetric channels that could be used to analyze the asymmetry of the neural representations of pitch across hemispheres was obtained.

Using this set of selected channels, hemispheric asymmetry was assessed by analyzing the mean ERP amplitudes in the time window between bilaterally symmetric channels. Further evaluation was performed to determine, whether differences in the mean ERP amplitude between bilaterally symmetric channels were asymmetric for pitch. For example, the amplitude was greater in the left than in the right channels for a lower pitch, and vice versa for a higher pitch. Therefore, a paired t-test (after the Kolmogorov–Smirnov test for normality) was performed to assess any significant differences (p < 0.05) in the mean ERP amplitude between each pair of bilaterally symmetric channels for each pitch in each group.

Finally, the time at which the ERP amplitudes became discriminative according to pitch was examined. The time window for the analysis was determined based on maximum discrimination; consequently, the time at which the ERP amplitudes became discriminative was identified. A one-way ANOVA was performed to test for any significant difference in the mean ERP amplitude among pitches in an optimal time window. For each group, the earliest time window in which significant differences (p < 0.05) were observed in more than half of the selected channels was identified. To avoid the multiple comparison problem, the false discovery rate (FDR) was applied to all relevant analyses.

## Results

In summary, first, the neural correlates of pitch in ERP amplitudes were investigated. Upon finding ERP correlates of pitch, whether these were presented differently between the hemispheres was examined. Second, the neural correlates of pitch were compared between the different brain areas. Finally, whether the neural correlates of pitch were more salient in the MT group than in the NT group was investigated. In addition, the behavioral differences between the MT and NT groups were investigated prior to identifying these neural correlates.

### Musically-trained individuals exhibit superior pitch discrimination

The MT group demonstrated superior performance than the NT group in the pitch discrimination task. The mean (± standard deviation) number of “correct” blocks, in which a participant correctly counted the number of target pitches in the block (Supplementary Table 1), was significantly larger in the MT group than in the NT group (12.5 ± 1.5 vs 3.3 ± 2.03, p < 0.0001, Wilcoxon rank sum test).

### Bilateral frontotemporal ERP waveforms show neural correlates of pitch in both MT and NT groups

**Fig. 2:**
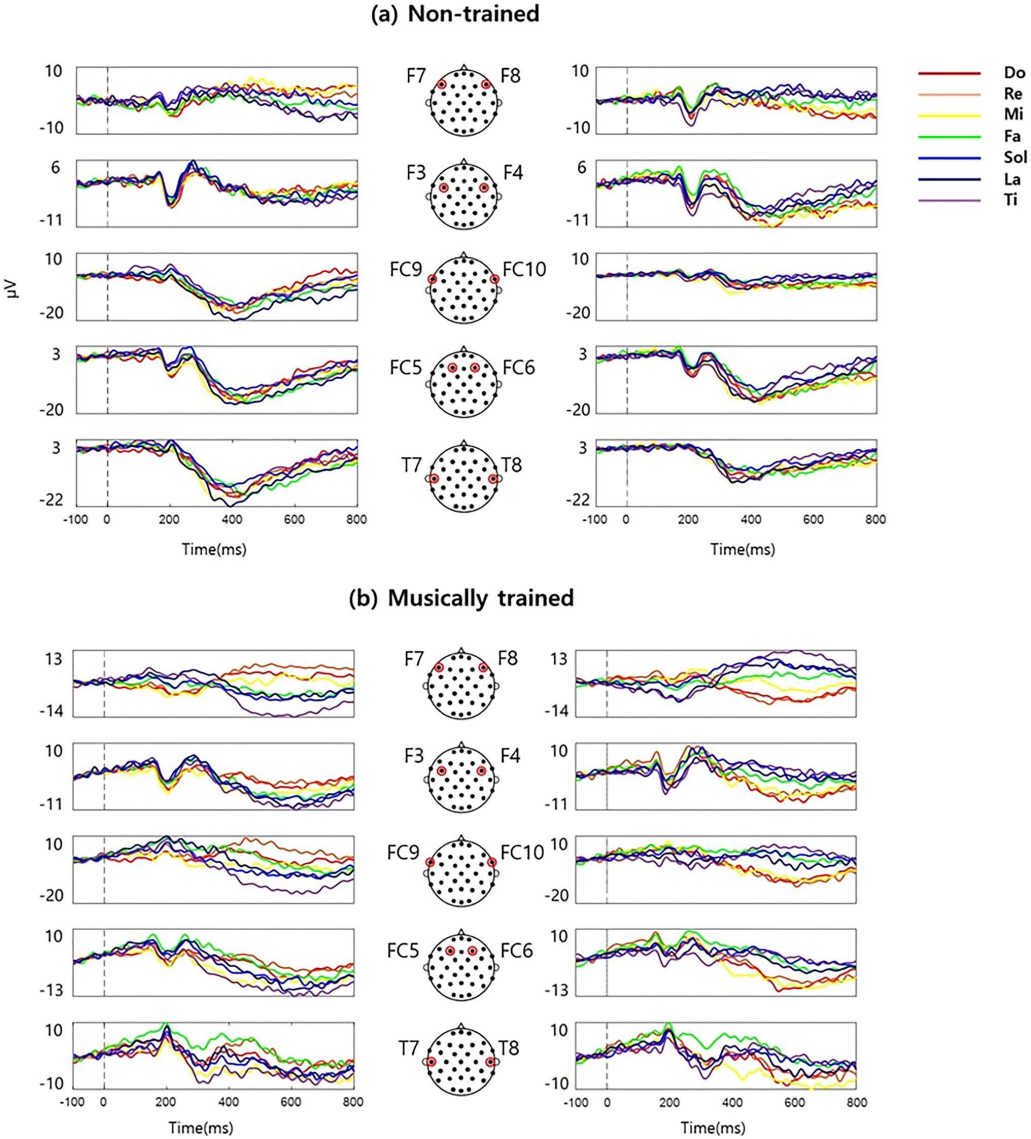
ERP patterns in response to different pitches. The ERP amplitudes from 100 ms before stimulus onset to 800 ms after stimulus onset at ten bilateral channels are presented for the non-trained (**a**) and musically-trained groups (**b**). The left and right columns of ERP graphs in each group correspond to the left and right hemispheric EEG channels, respectively. The color of each ERP graph indicates corresponding pitch stimulus (see legend). The ERP amplitudes represent the group average. ERP: event-related potential

To identify neural representations of pitch in each group, ERP waveforms at each EEG channel (100 ms before stimulus onset to 800 ms after stimulus onset, corresponding to individual pitches) were assessed (Supplementary Fig. 1). The ERP waveforms revealed pitch-discriminative patterns, where ERP amplitudes varied with pitch. This pitch discrimination in the ERP amplitudes in different time windows was assessed using the aforementioned separability index. The results revealed that the greatest separability among pitches in the window between 540 and 640 ms after stimulus onset (Supplementary Fig. 2). Using ERP data from this time window, one-way ANOVA was used to select five pairs of bilaterally symmetric channels that demonstrated a significant difference in mean amplitudes for the following pitches: F7-F8, F3-F4, FC9-FC10, FC5-FC6, and T7-T8. The selected pairs were distributed over the frontocentral and temporal areas rather than over the parietal areas (Fig. 2A). The ERP waveforms in these channels clearly exhibited discriminative patterns for different pitches (Fig. 2B, C).

### ERP amplitudes vary with pitch in an anti-symmetric manner between hemispheres

**Fig. 3:**
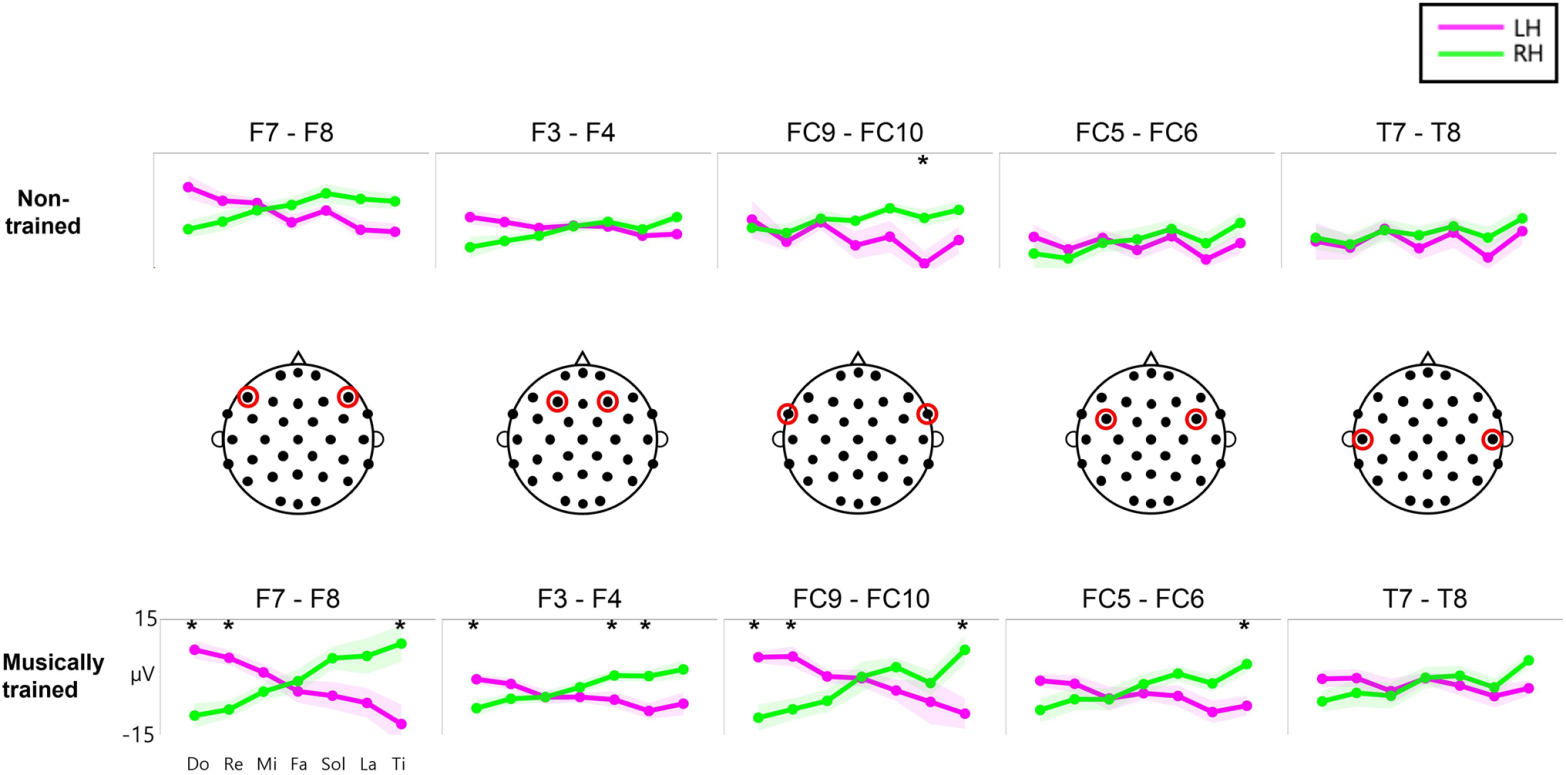
Anti-symmetric ERP amplitude variations with pitch between hemispheres. The variation of the ERP amplitudes that averaged within 540–640 ms after stimulus onset over seven pitches is illustrated for five different pairs of bilateral EEG channels in each group (top: non-trained group; bottom: musically-trained group). The line represents the group average of ERP amplitudes, and the shading represents standard error of mean. The black star denotes a significant difference in the ERP amplitudes between the left and right hemispheres (paired t-test, p < 0.05, FDR correction). The red circles on the topographs in the middle indicate the channel location for each column. LH: left hemisphere; RH: right hemisphere; ERP: event-related potential

We investigated whether the patterns of ERP amplitude varying with pitch differed between hemispheres. Anti-symmetric patterns were found between hemispheres (Fig. 3) by examining the variations of the mean ERP amplitudes within the time window of analysis with pitch for each pair of bilaterally matched channels (Supplementary Table 2 presents the mean ERP amplitude values), Although the degree of change in the amplitude according to pitch was apparently different across the channels, all the channels demonstrated a marked tendency for the mean amplitudes to become more negative as pitch increased (from C4– B4) in the left hemisphere (i.e., F7, F3, FC9, FC5, and T7); conversely, they became more negative as the pitch decreased in the right hemisphere (i.e., F4, F8, FC6, FC10, and T8) (Fig. 3).

### ERP amplitudes linearly correlate with pitch

The ERP amplitudes changed linearly with pitch, and the degrees of increase or decrease in the amplitudes appeared to vary both across the channels and across the groups (Fig. 3). We fitted linear regression models between the mean amplitudes and pitch for each channel to examine this apparent linear change in ERP amplitudes with pitch. The pitch value was assigned an equal space of one to seven for C4–B4, respectively. The slope of each linear fit for each channel was estimated for each group (Fig. 5A, Supplementary Table 3 and 4). All the slopes were significant (one-sampled t-test, p < 0.05, FDR correction); negative and positive for the left and right hemispheres, respectively, confirming that the ERP amplitudes changed linearly with pitch.

### Frontal areas demonstrate greater hemispheric differences than parietal areas

Symmetrical patterns appeared most prominently in the channel pairs in the frontotemporal area; therefore, the frontotemporal and parietal channel pairs, which are symmetric with respect to the coronal plane, were compared for validation.

**Fig. 4:**
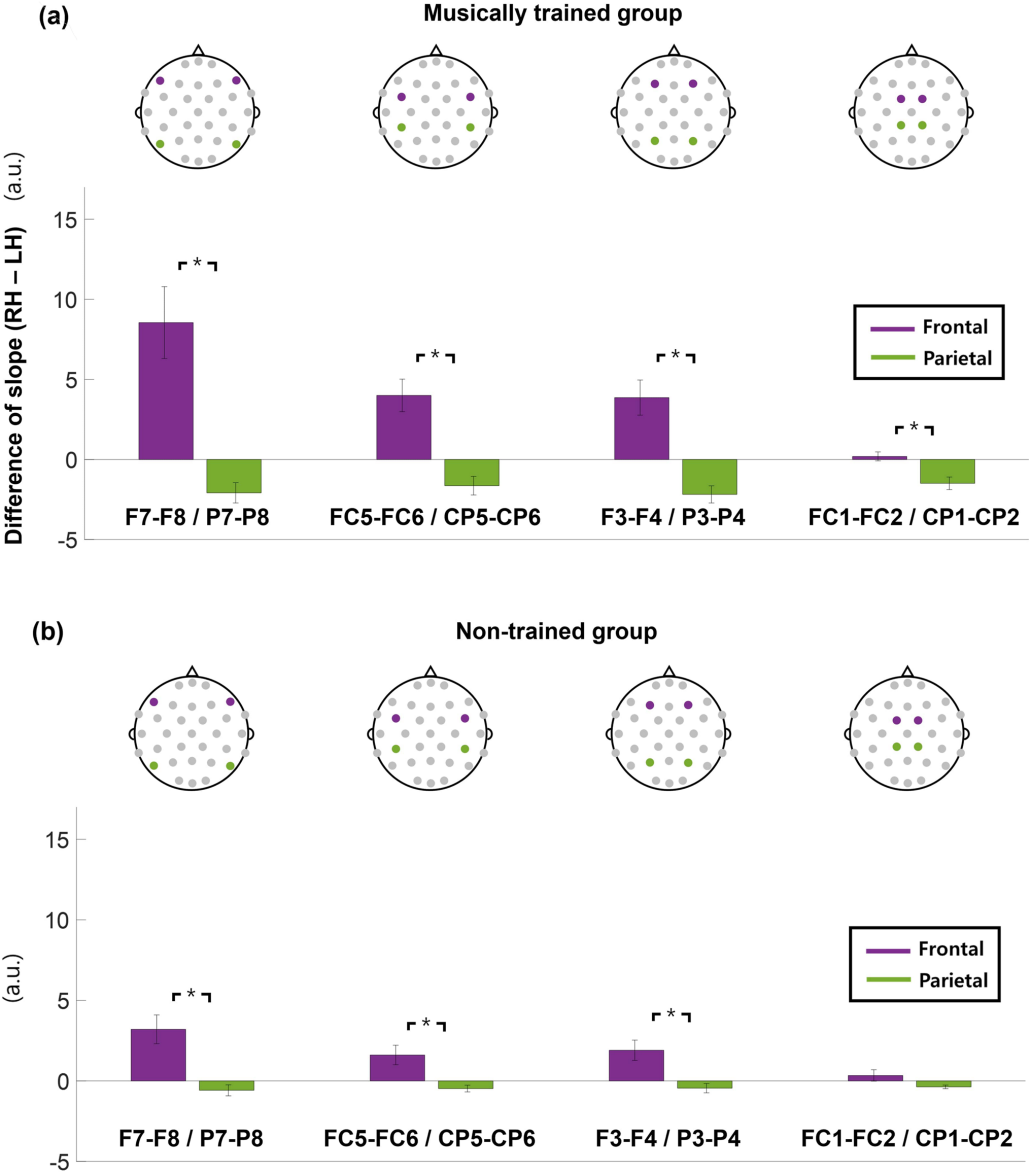
Comparison of hemispheric asymmetry measurements between frontal and parietal areas. Hemispheric asymmetry is measured according to a difference of fitted slopes between bilateral EEG channels (RH-LH). The montages locate dyadic bilateral channel pairs across frontal and parietal areas, which are symmetrical with respect to the coronal plane (purple: frontal area; green: parietal area). Each bar graph represents the average of values of hemispheric asymmetry measurements. The error bar indicates the standard error of mean (SEM). The vertical axis represents the difference of fitted slopes (arbitrary units). **a** Musically-trained group. **b** Non-trained group. (*: p < 0.05, FDR correction). LH: left hemisphere; RH: right hemisphere; FDR: false discovery rate

Four pairs of bilateral channels in the frontal (F7-F8, FC5-FC6, F3-F4, and FC1-FC2) and parietal areas (P7-P8, CP5-CP6, P3-P4, and CP1-CP2) were respectively collected. Subsequently, the bilateral difference of fitted slopes (i.e., the difference between the slopes of the corresponding left and right channels) in each pair was calculated. The results revealed that every frontal pair exhibited a significantly larger bilateral difference in slope than its parietal counterpart for each group (Wilcoxon signed-rank test, p < 0.05, FDR correction) (Fig. 4). The overall ERP pattern was also illustrated in Supplementary Fig. 1.

### Musical training strengthens linear relationships between ERP amplitudes with pitch

**Fig. 5:**
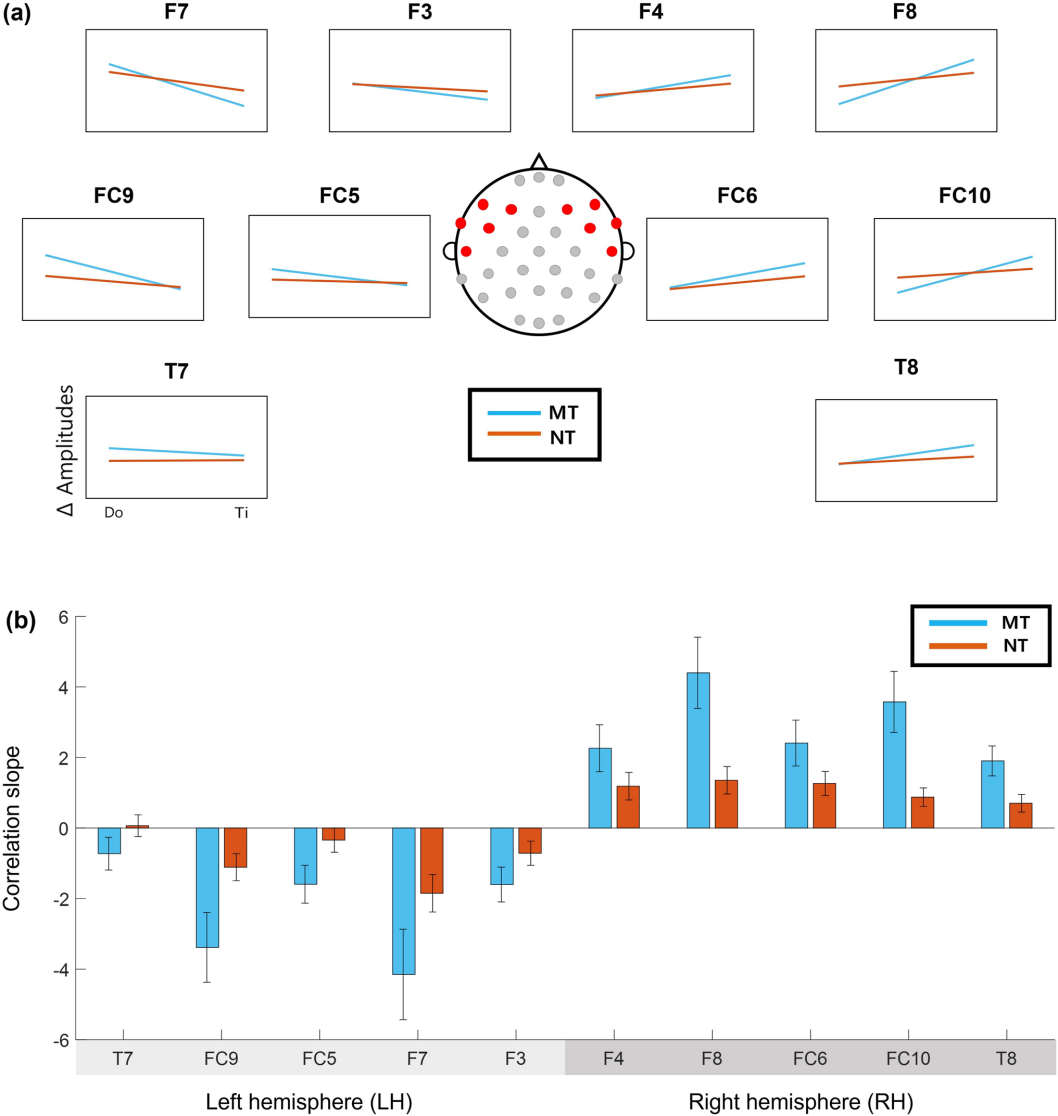
The slope of linear models in each group. **a** The slopes of linear models between the mean amplitudes and pitch for each channel are presented (cyan: musically-trained group; orange: non-trained group). The slope shows the average of individual linear models fitted to the data of 10 subjects in each group. The red dots on the montage indicate corresponding channel locations. The horizontal axis represents pitch from “Do” to “Ti,” and the vertical axis represents the change of the ERP amplitude from pitch by a linear model (elucidated in the text). All lines are adjusted to take x-intercept at “Fa” for easy comparison. **b** The slope of a linear model fitted to the mean ERP amplitude against pitch is displayed at each channel for each group. The error bar on each bar graph represents the standard error of mean (SEM) for each group at each channel. The graphs are bilaterally arranged by channel locations from left to right. NT: non-trained group; MT: musically-trained group; ERP: event-related potential

The effect of musical training on neural representations of pitch was examined. The slopes of the linear regression models were compared between the MT and NT groups (Fig. 5B). The MT group exhibited stiffer slopes than the NT group for every channel in both hemispheres. There were no significant channels after FDR correction. A correlation analysis between corrected block counts and the slopes of linear fit across individual participants was conducted in each channel. We found significant correlations at 7 frontal channels (Supplementary Table 5).

## Discussion

Neural coding of auditory stimulus frequency is well documented; however, the cortical signals for frequency of pitch are yet to be comprehensively elucidated. The present study investigated cortical representations of musical pitch by analyzing human EEG. Therefore, first, we addressed whether it was possible to find neural correlates of pitch in ERPs. We found that the ERP amplitudes appearing approximately 400 ms after stimulus onset while participants perceived different pitches on a musical scale demonstrated pitch correlations. Second, we examined if these ERP correlates of pitch were different between hemispheres according to hemispheric asymmetry in musical processing. We found inter-hemispheric anti-symmetric ERP patterns; namely, the left and right hemispheric ERP amplitudes decreased and increased, respectively, as the pitch increased. Notably, the spatial patterns of these correlations between the ERP amplitudes and pitch frequency exhibited hemispheric symmetry. Third, we investigated the brain areas wherein these correlations were more pronounced. We found that these patterns appeared most prominently in the frontotemporal area. We further examined this observation by comparing the slopes of linear regression models fitted to the ERP amplitudes and pitch between the frontotemporal and parietal areas, which are symmetric with respect to the coronal plane, and verified that the slopes were steeper in the frontotemporal area than in the parietal area. Fourth, we explored whether neural correlates of pitch were more salient in the MT group than in the NT group. Although the behavioral performance of pitch processing was significantly different between the MT and NT groups, ERP correlates of pitch were observed in both groups. Moreover, the MT group exhibited marginally stronger correlations between the ERP amplitudes and pitch frequency than the NT group. These findings indicate cortical representations of musical pitch in the ERP patterns.

### Anti-symmetric patterns of neural correlates of pitch across hemispheres

Although existing studies report that cortical representations of pitch could differ between hemispheres, the apparent anti-symmetric patterns of linear relationship between ERP amplitudes and pitch in our study were rather unexpected [1, 8]. Therefore, we posit a possible explanation for the observed ERP patterns. In the spatial-musical association of response codes (SMARC) effect the human mind maps higher- and lower-frequency sounds toward the top or right and bottom or left, respectively, demonstrating a natural internal pitch-space relation [45, 46, 52, 57, 58]. The neural correlates for the SMARC effect remain unknown; however, the analogous spatial-numerical association of response code (SNARC) effect has been widely studied.

In the SNARC effect, smaller and larger numbers are mapped to the left and right, respectively, along the mental number line [29, 64]. Studies have reported that neural processing underlying the SNARC effect involves contrasting neural activities in the parietal cortex. Specifically, larger and smaller numbers are preferentially processed in the left and right parietal cortical areas, respectively, reflecting our results in pitch perception. The anti-symmetric patterns across hemispheres in the neural correlates of the SNARC effect suggest that the similar antisymmetric patterns observed in the current study may reflect SMARC effect-related neural processing.

### Cortical representations of pitch in frontotemporal areas

In this study, the neural correlates of pitch were prominently observed in the bilateral frontotemporal areas. Frontotemporal cortical areas are key in musical information processing, as reported in several amusia studies [61, 63]. Tissieres et al. [63] reported that patients with lesions in the left temporo-parieto-frontal cortex lost their sound localization ability [63]. Moreover, some functional magnetic resonance imaging studies have reported that the IFG and left superior frontal gyrus were activated when subjects inductively inferred spatial information. In addition, the left IFG was activated when extracting spatial information rules [20]. These results support our findings that hemispheric asymmetry in the frontotemporal area may related to pitch information processing.

### Slow ERP waveforms associated with pitch discrimination

In the present study pitch-discriminative patterns were observed to apparently begin than the typical latency of most AEPs. Particularly, mismatch negativity (MMN) in ERPs elicited by the recognition of differences among various musical components, including pitch, appeared approximately 200 ms after onset [31]. However, MMN reflects the detection of a difference in a deviated musical component from a standard component, based on the oddball paradigm. Moreover, participants in our study discriminated one out of several pitches from randomly presented auditory stimuli. This pitch discrimination task possibly involved higher-level cognitive processing than simple detection represented by AEPs; therefore, the latency of pitch-discriminative patterns is longer than that of AEPs. In addition, a recent study has reported that N1, P2, and early right-anterior negativity components are related to the degree of the scale of probe tones [62]. The results revealed distinct ERP patterns according to pitch in N1, P2, and especially ERP components at 400–600 ms, similar to the discriminative ERP patterns observed in our study. Moreover, the neurocognitive model for music perception proposed by Koelsch & Siebel [36] illustrated that N400 and N5 components are related to meaning,for example, “bright,” “bright-rough,” “rough,” or “dull,” which are conveyed through a single tone. Further, the late positive and P600 components are related to structural reanalysis [36]. This possibly implied that the participant reconstructed the relative meaning of perceived pitch on a musical scale after listening to pitch stimuli. The ERPs at these channels T7 and T8 were selected as our region of interest and dealt with in all our main analysis except the comparison between frontal and parietal areas.

### Musical training effects

Our behavioral data indicated that the MT group outperformed the NT group regarding processing of pitch information. Pitch-discriminative ERP patterns appeared in both groups; however, they were more pronounced in the MT group. This suggests that, though neural representations of pitch are commonly present regardless of musical training, cortical pitch information processing is more prominent in the MT group. In Fig. 5B, we couldn't observe the significant difference in the slope of linear models between groups. We speculated that it is due to the small sample size. However, we observed the clear tendency that MT group has more steeper slopes. Several studies have reported superior pitch discrimination due to musical training [22, 24, 26]. Musical training increases the musical performance-associated brain plasticity [59, 60]; therefore, our results indicate that musical training may further enhance neural processing related to the spatial association of pitch.

### Limitations and further work

This study presented hemispheric asymmetric patterns that were illustrated only in simple C major scales. Furthermore, verification is needed to determine whether these asymmetric patterns stemmed from the rearrangement of single tones of pitch frequency in the inner mind or from the recognition of single tones. Therefore, the investigation of neural correlates with each semi-tone of pitch frequency (for example, C, C#, D, and D# on the C Major scale) should be conducted. In addition, the present study may not dissociate between pitch chroma and pitch height (i.e., F0). To address this, further studies should investigate whether our findings are valid for other octaves.

### Conclusions

This study provides evidence for the neural representation of pitch frequency through ERP analysis. In addition, it provided interesting hemispheric asymmetric patterns for pitch frequency, which is unprecedented, to the best of our knowledge. These findings provide a basis for musical brain–computer interface applications and evidence for understanding the cognitive process of pitch frequency. The distinctive ERP patterns in response to each single tone of pitch chroma found in this study indicate a possibility that similar patterns may appear when people imagine single tones. This possibility may offer opportunities to decode pitch information from EEG signals and consequently, help build a brain-computer interface to produce pitch sounds from brain activity. Our present study will serve as a springboard for our follow-up study to investigate this possibility.

## Supplementary Information

Below is the link to the electronic supplementary material.

**Supplementary file 1: Supplementary Fig. 1.** ERP patterns in response to different pitches. The ERP amplitudes from 100 ms before stimulus onset to 800 ms after stimulus onset at all channels. ERP patterns are presented for the non-trained (a) and musically-trained groups (b). The color of each ERP graph indicates corresponding pitch stimulus (see legend). The ERP amplitudes represent the group average. ERP: event-related potential. (JPG 396 kb)

Supplementary file 2: Supplementary Fig. 1b (JPG 402 kb)

**Supplementary file 3: Supplementary Fig. 2.** Separability. The Separability from stimulus onset to 800 ms after stimulus onset. Separability was calculated in 100 ms window for each timepoint. Bold line is averaged separability and gray shade is standard error of the mean(SEM). (JPG 34 kb)

**Supplementary file 4: Supplementary Table 1**. Behavior result. The number of “correct” blocks, in which a participant correctly counted the number of target pitches in the block. (JPG 60 kb)

**Supplementary file 5: Supplementary Table 2.** Mean amplitudes of ERP in ROI channels for each pitch. Mean ERP amplitudes within the time window of analysis with pitch for each pair of bilaterally matched channels. (JPG 244 kb)

**Supplementary file 6: Supplementary Table 3.** The slope of each linear fit each pair of bilaterally matched channels for each group. All the slopes were significant in both groups(one-sampled t-test, p < 0.05, FDR correction). (JPG 60 kb)

**Supplementary file 7: Supplementary Table 4.** The correlation coefficient(r) and p-value of each linear fit each pair of bilaterally matched channels for each group(one-sampled t-test, p < 0.05, FDR correction). (JPG 187 kb)

**Supplementary file 8: Supplementary Table 5.** The correlation analysis between corrected block counts and the slopes of linear fit across individual participants was conducted in each channel(one-sampled t-test, p < 0.05, FDR correction). (JPG 39 kb)

## Data Availability

The datasets generated during and/or analyzed during the current study are available at https://www.unist-bci.com/datasets. If there are any problems in the data or code in the repository, please contact kthwork9934@gmail.com or spkim@unist.ac.kr.
