# Supplementary figures and images for: Cortical representation of musical pitch in event-related potentials
Source: Biomed Eng Lett. 2023 Apr 13;13(3):441–54. doi: 10.1007/s13534-023-00274-y (PMC10382469; doi:10.1007/s13534-023-00274-y)

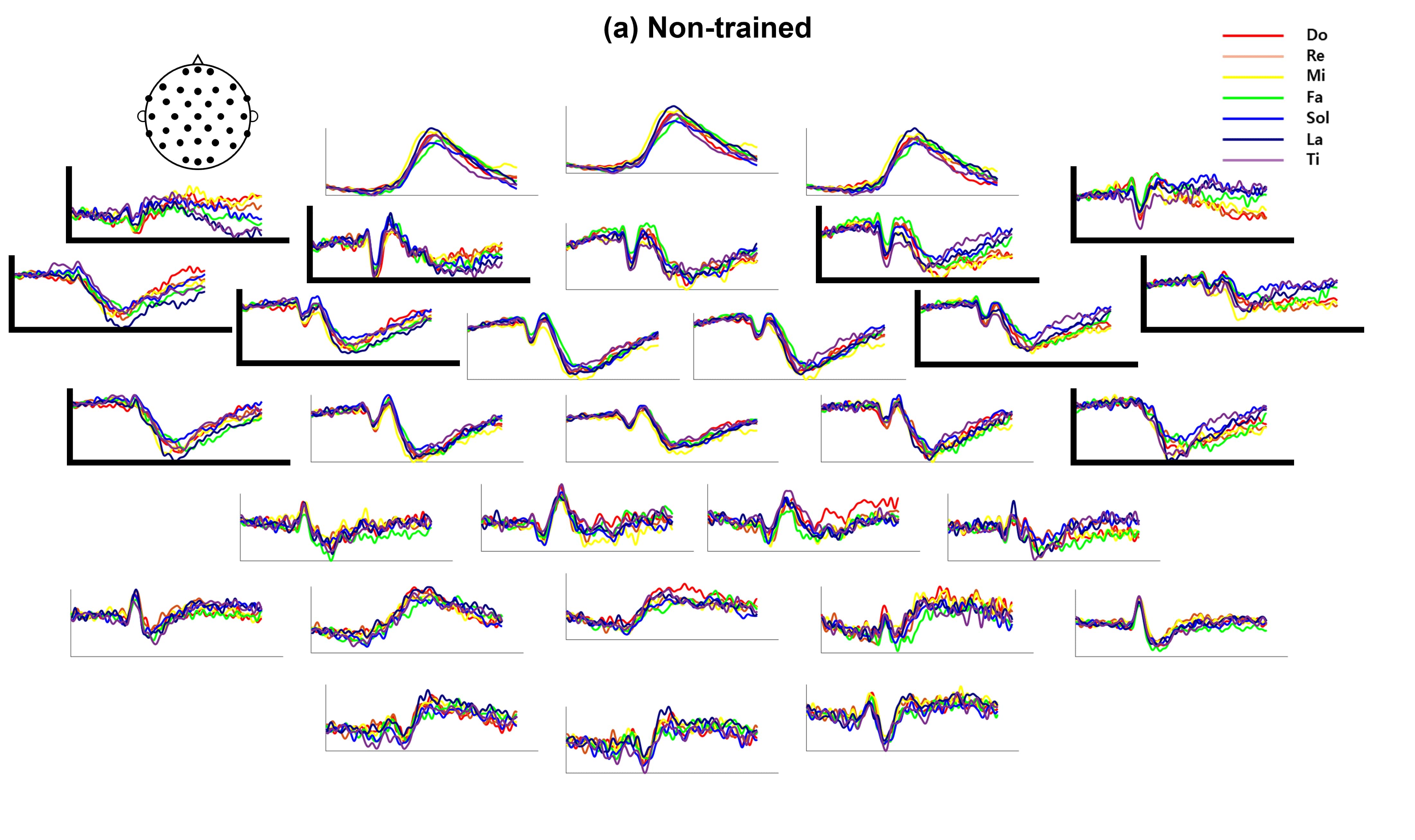

Supplement: Supplementary file 1 — Supplementary Fig. 1. ERP patterns in response to different pitches. The ERP amplitudes from 100 ms before stimulus onset to 800 ms after stimulus onset at all channels. ERP patterns are presented for the non-trained (a) and musically-trained groups (b). The color of each ERP graph indicates corresponding pitch stimulus (see legend). The ERP amplitudes represent the group average. ERP: event-related potential. (JPG 396 kb) [file 13534_2023_274_MOESM1_ESM.jpg]

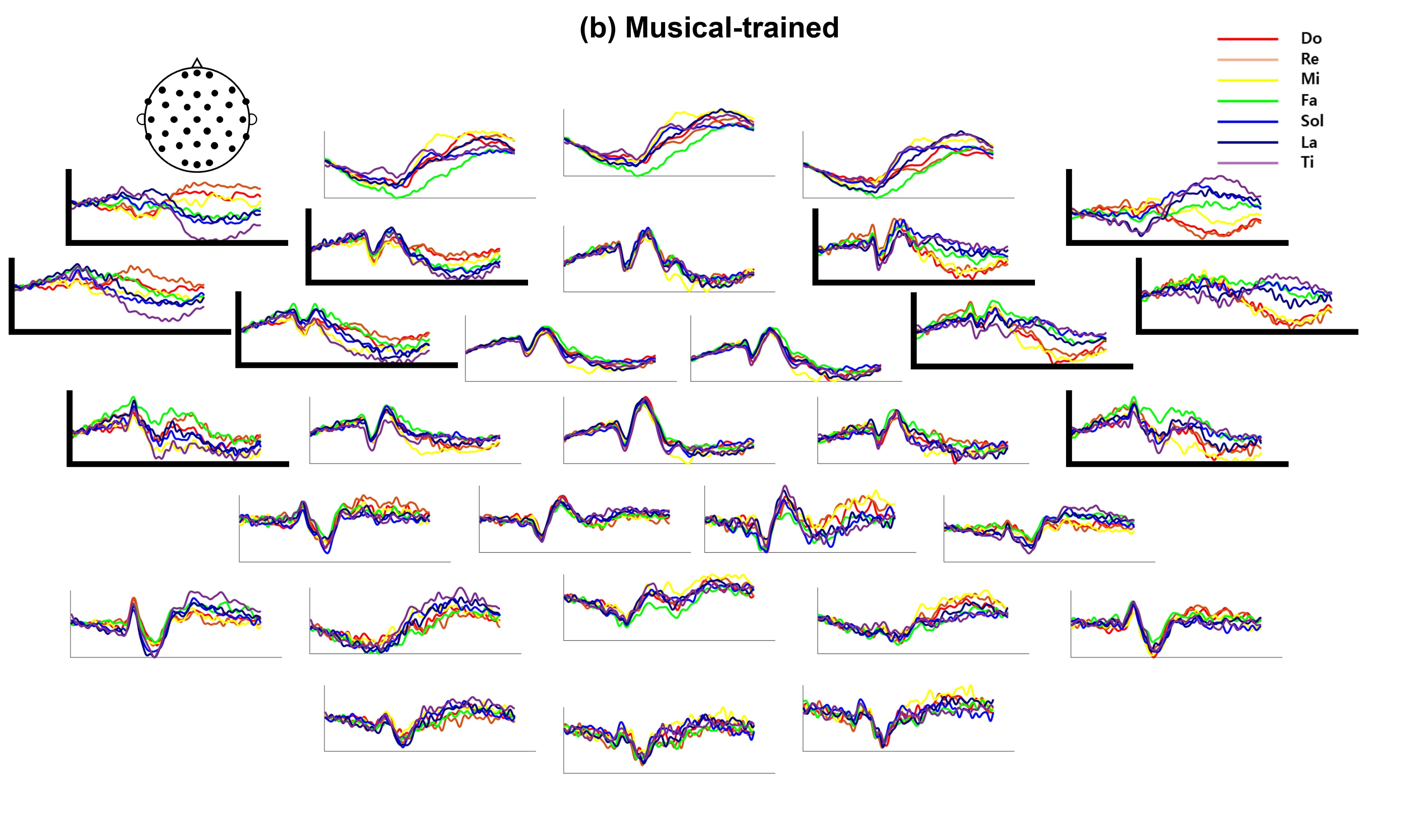

Supplement: Supplementary file 2 — Supplementary Fig. 1b (JPG 402 kb) [file 13534_2023_274_MOESM2_ESM.jpg]

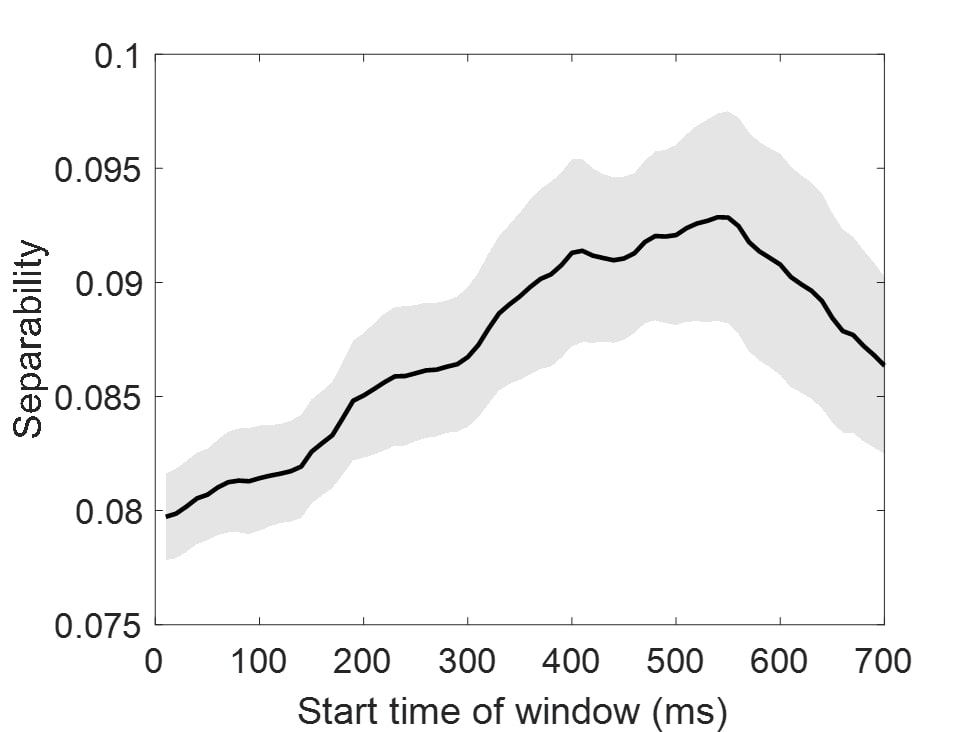

Supplement: Supplementary file 3 — Supplementary Fig. 2. Separability. The Separability from stimulus onset to 800 ms after stimulus onset. Separability was calculated in 100 ms window for each timepoint. Bold line is averaged separability and gray shade is standard error of the mean(SEM). (JPG 34 kb) [file 13534_2023_274_MOESM3_ESM.jpg]

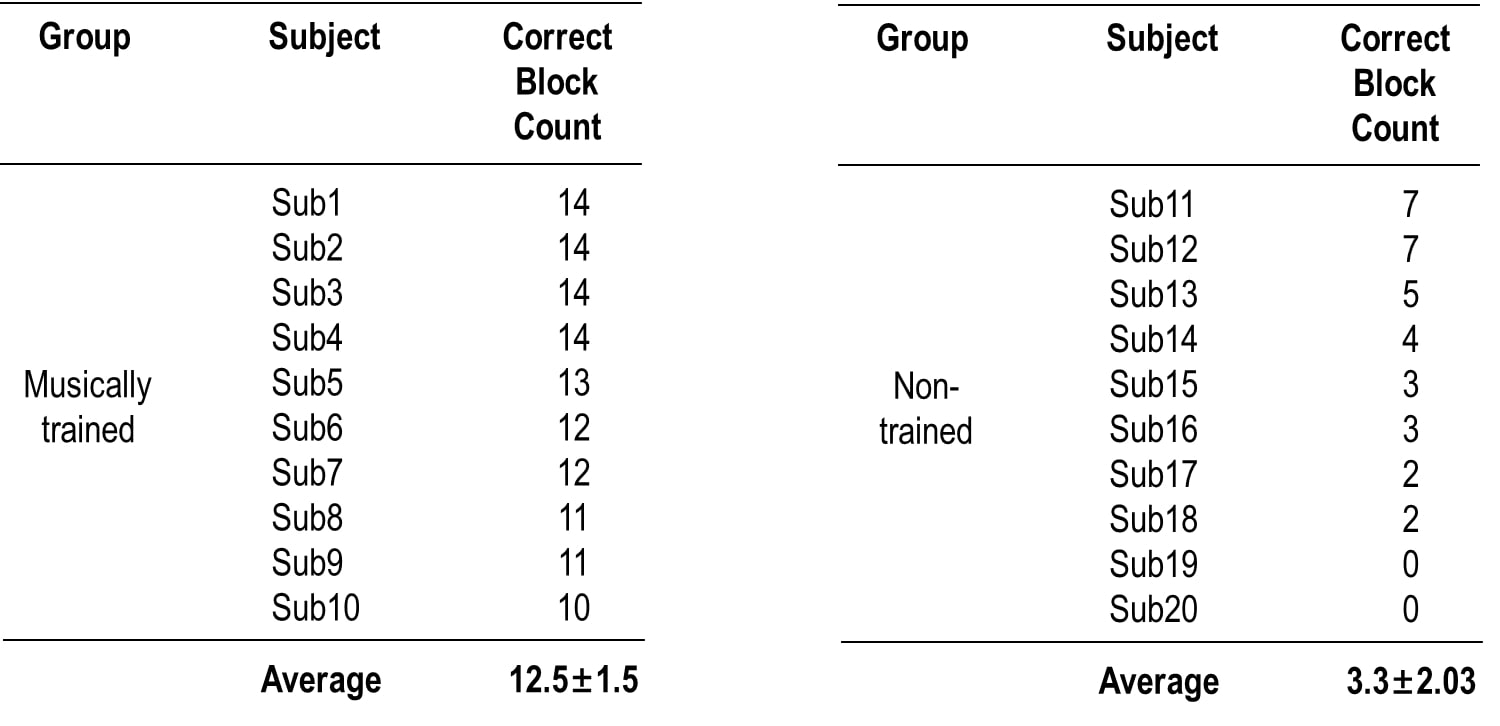

Supplement: Supplementary file 4 — Supplementary Table 1. Behavior result. The number of “correct” blocks, in which a participant correctly counted the number of target pitches in the block. (JPG 60 kb) [file 13534_2023_274_MOESM4_ESM.jpg]

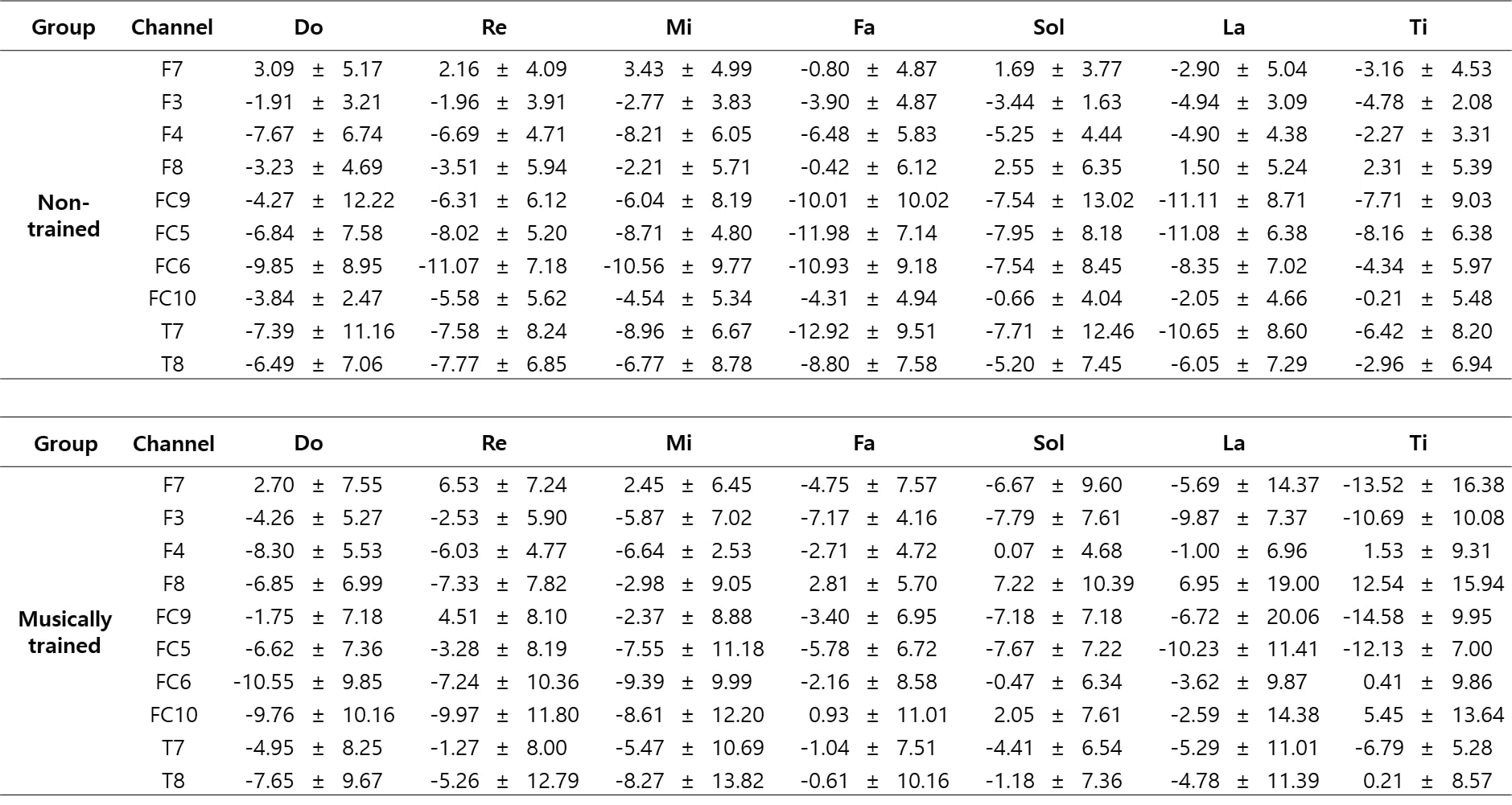

Supplement: Supplementary file 5 — Supplementary Table 2. Mean amplitudes of ERP in ROI channels for each pitch. Mean ERP amplitudes within the time window of analysis with pitch for each pair of bilaterally matched channels. (JPG 244 kb) [file 13534_2023_274_MOESM5_ESM.jpg]

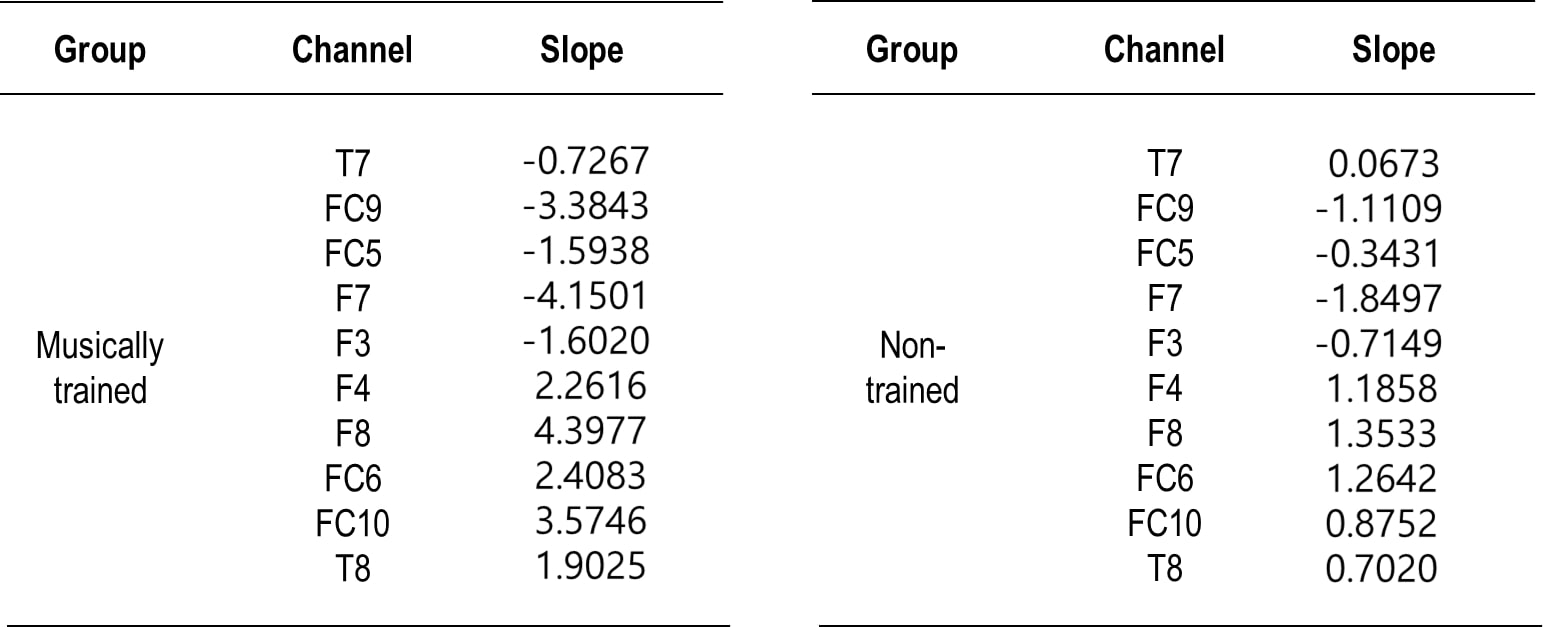

Supplement: Supplementary file 6 — Supplementary Table 3. The slope of each linear fit each pair of bilaterally matched channels for each group. All the slopes were significant in both groups(one-sampled t-test, p < 0.05, FDR correction). (JPG 60 kb) [file 13534_2023_274_MOESM6_ESM.jpg]

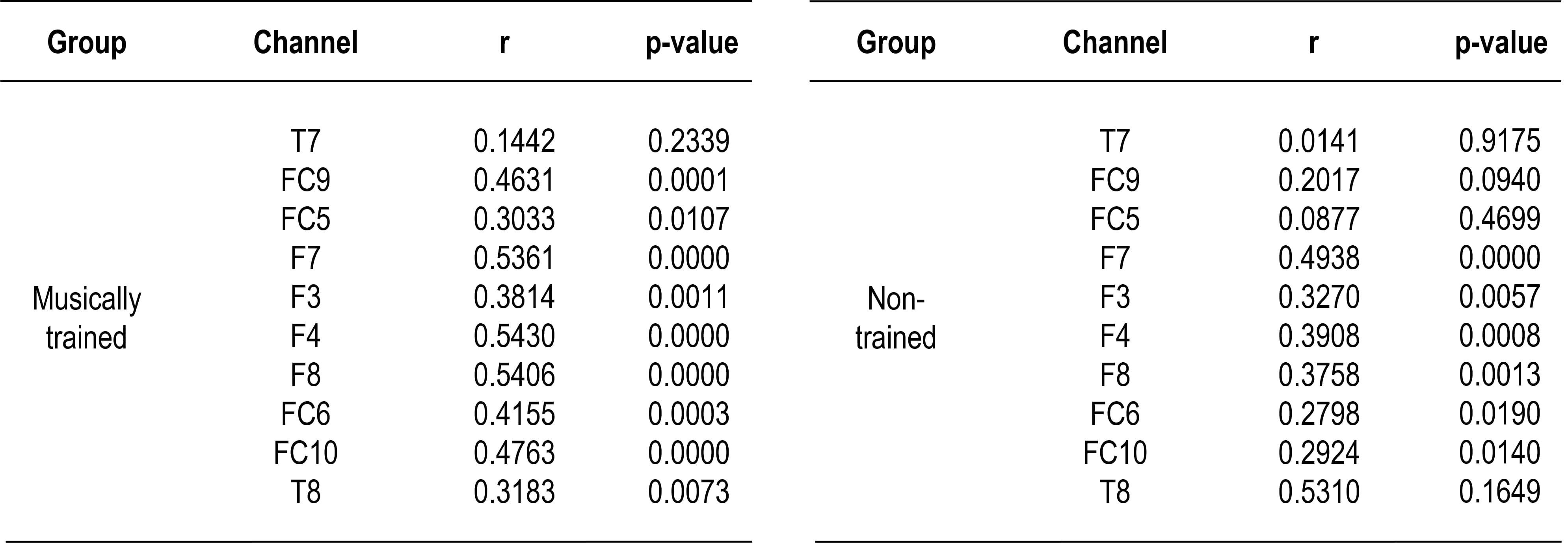

Supplement: Supplementary file 7 — Supplementary Table 4. The correlation coefficient(r) and p-value of each linear fit each pair of bilaterally matched channels for each group(one-sampled t-test, p < 0.05, FDR correction). (JPG 187 kb) [file 13534_2023_274_MOESM7_ESM.jpg]

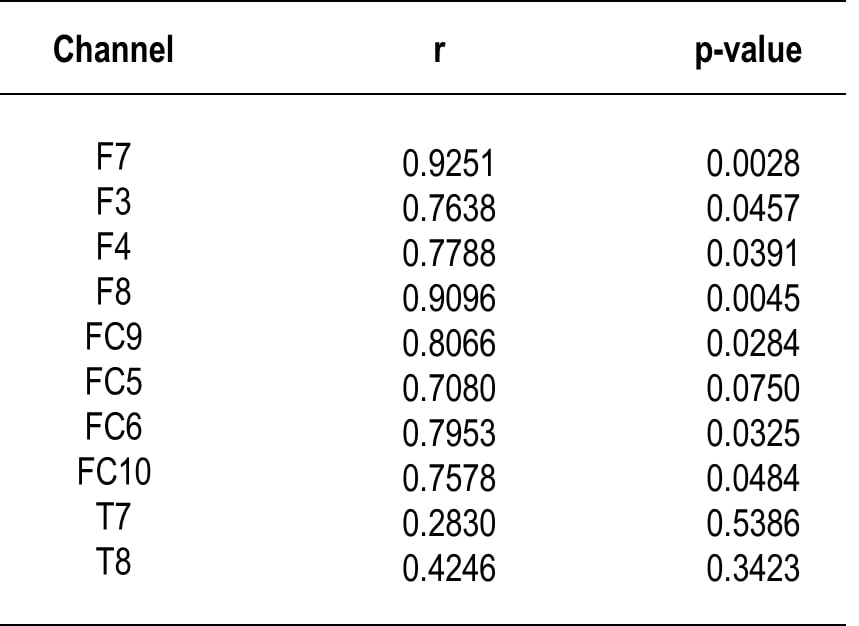

Supplement: Supplementary file 8 — Supplementary Table 5. The correlation analysis between corrected block counts and the slopes of linear fit across individual participants was conducted in each channel(one-sampled t-test, p < 0.05, FDR correction). (JPG 39 kb) [file 13534_2023_274_MOESM8_ESM.jpg]
